# Flagellate erythema and breast infiltration

**DOI:** 10.1016/j.jdcr.2025.07.026

**Published:** 2025-08-20

**Authors:** Laura Funaro, Lucas Boussingault, Farida Benhadou, Mohammad Yassine Chérif

**Affiliations:** a Department of Rheumatology, Erasme University Hospital Université Libre de Bruxelles (ULB), Brussels, Belgium; b Department of Dermatology, Erasme University Hospital Université Libre de Bruxelles (ULB), Brussels, Belgium

**Keywords:** cancer associated myositis, connective tissue disease, dermatomyositis, flagellate erythema, immunology, rheumatology

## Case presentation

**Fig 1:**
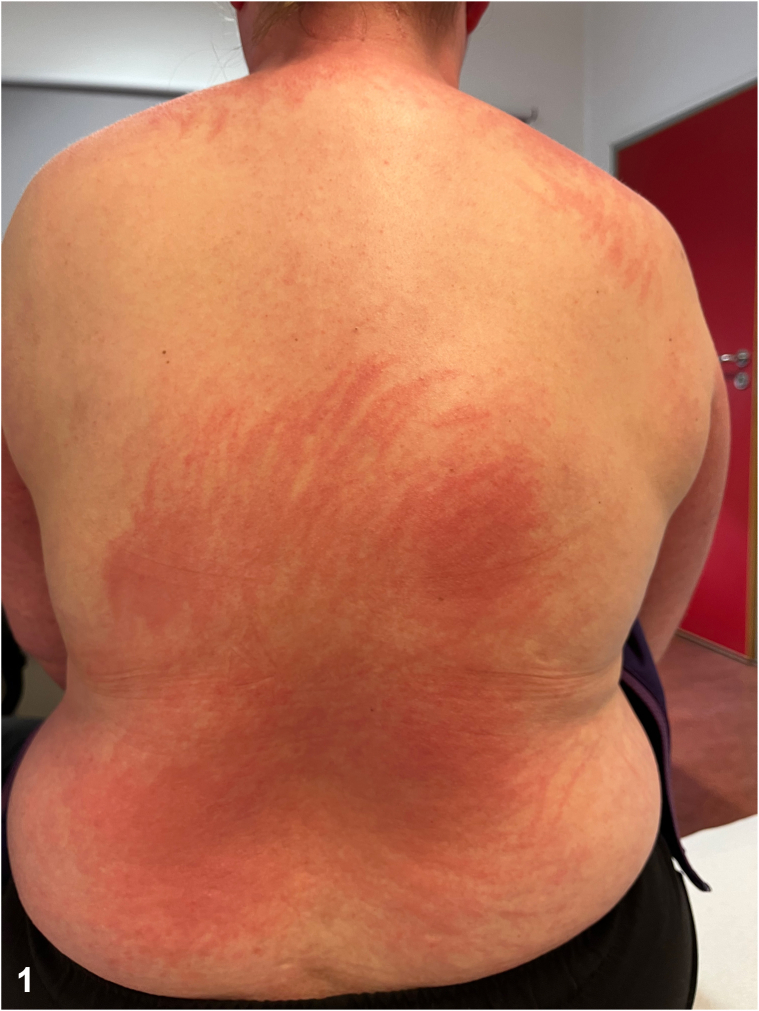

**Fig 2:**
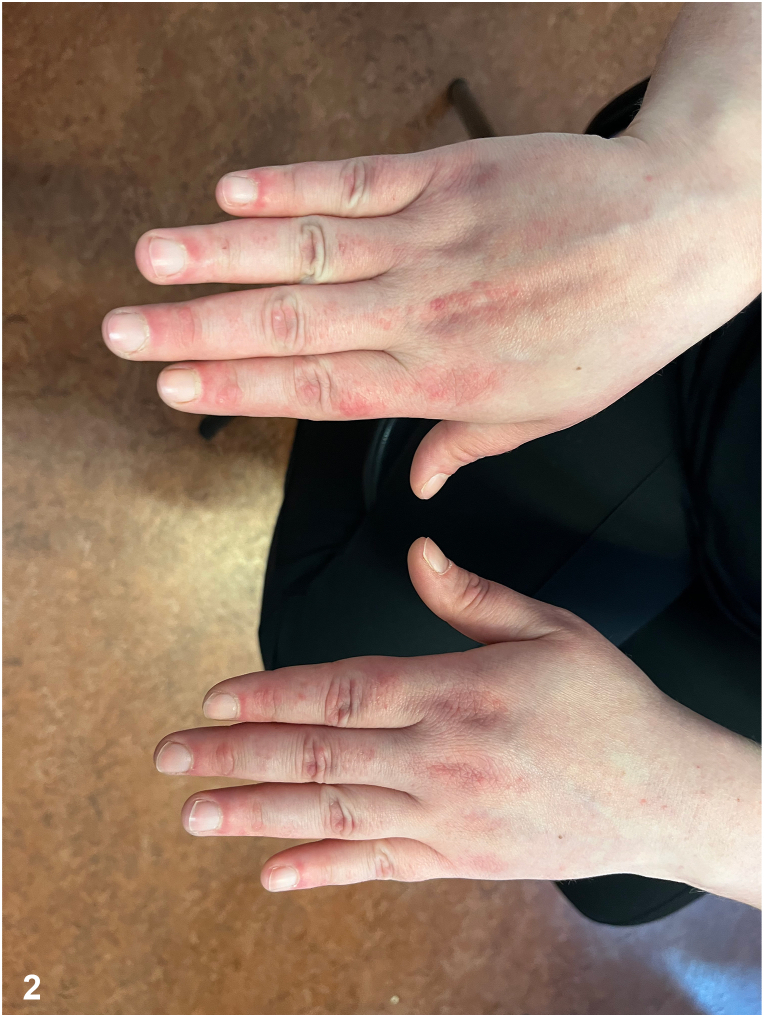

A 36-year-old woman presented with a 2-month history of a pruritic skin rash and right breast pain that had developed simultaneously. The rash were distributed across her back, face, and all 4 limbs. Physical examination revealed a flagellate erythema on the back (Fig 1), a painful periungual erythema, an erythematous area involving the upper back, shoulders and back of the neck, small red flat papules on extensor surfaces of the hands (Fig 2), a violaceous rash over the lateral hip and livedo reticularis. A painful infiltration was noted in the upper outer quadrant of the right breast. Pulmonary clinical examination and muscle strength were unremarkable. Laboratory tests showed the presence of antinuclear antibodies with a fine speckled nuclear pattern at a titer of 1:80, without creatine kinase or aldolase elevation.

**Question 1: Given the suspected diagnosis and the immunofluorescence pattern, which type of antibodies would most likely be detected in our patient?**

A. Anti-TIF1γ
B. Anti-HMGCR
C. Anti-Jo1
D. Anti-cN1A
E. Anti-NXP-2

**Answers:**

A. Anti TIF1γ – Correct. Anti-TIF1γ antibodies can be found in cancer associated myositis (up to 60% to 80%) and typically express a fine speckled nuclear pattern in indirect immunofluorescence.^1^
B. Anti-HMGCR – Incorrect. This antibody is primarily associated with immune-mediated necrotizing myopathy (IMNM). Although the clinical presentation of IMNM can resemble a dermatomyositis-like phenotype, the cancer risk in anti-HMGCR positive patients appears to be mild, and is more frequently reported in seronegative IMNM.^2^
C. Anti Jo1 – Incorrect. This antibody is characteristic of myositis associated with antisynthetase syndrome and dermatomyositis. However, these antibodies typically express a cytoplasmic fine speckled immunofluorescence pattern.
D. Anti cN1A – Incorrect. This antibody may be found in inclusion body myositis that doesn’t match with the clinical picture. Furthermore, no association with malignancy has been described.
E. Anti-NXP2 – Incorrect. While this antibody is indeed described in cancer-associated myositis, neither the clinical examination showing predominant muscle involvement nor its immunofluorescence pattern rather corresponds to multiple nuclear dots.^3^

**Question 2: Antibodies with specificity against TIF1γ antigens were found in our patient. In this context, which additional examination(s) would be most appropriate to investigate the underlying cause of this pathology?**

A. Computed tomography (CT) the chest, abdomen and pelvis
B. Complementary gynecological (pelvic ultrasonography and mammography) assessment
C. Pulmonary function tests (PFTs), including diffuse capacity of the lungs for carbon monoxide measurement
D. Biopsy of the flagellate erythema
E. Electroneuromyography

**Answers:**

A. CT the chest, abdomen, and pelvis – Correct. In the case of seronegative dermatomyositis or associated with the presence of anti-NXP2 or anti-TIF1γ, a systematic screening for cancer must be systematic and this imaging is essential to screen an underlying tumor.
B. Complementary gynecological (pelvic ultrasonography and mammography) assessment – Correct. Since breast and ovarian cancers are among the most common malignancies associated with dermatomyositis in women, these additional investigations are mandatory in such cases. In fact, further work-ups including a mammogram, breast ultrasound with needle biopsy, and a positron emission tomography-CT revealed a nonmetastatic right breast cancer in our patient.
C. PFTs, including diffuse capacity of the lungs for carbon monoxide measurement – Incorrect. PFTs and diffuse capacity of the lungs for carbon monoxide is useful for detecting ventilatory disorders or diffusion impairments, particularly in cases of interstitial lung disease associated with myositis but they do not contribute to identifying the underlying cause.
D. Biopsy of the flagellate erythema – Incorrect. It can help confirm the diagnosis in case of atypical features or assist in the differential diagnosis by revealing interface dermatitis, but it does not establish the disease etiology. In our case and as mentioned above, given the painful breast infiltration, imaging and guided biopsy of the mass led to the diagnosis of nonmetastatic breast cancer.
E. Electroneuromyography – Incorrect. Although useful in assessing muscle involvement in myositis by identifying a myogenic pattern and helping to pinpoint the most “rewarding” muscles for biopsy, electroneuromyography is not suitable for detecting an underlying malignancy.

**Question 3: Which of the following conditions can also present with flagellate erythema?**

A. Consumption of shiitake mushrooms
B. Bleomycin induced toxicity
C. Still disease
D. Pellagra
E. Sarcoidosis

**Answers:**

A. Consumption of shiitake mushrooms – Correct. It is a toxic reaction resulting from the ingestion of undercooked shiitake mushrooms and it may present as flagellate erythema. The condition is likely triggered by lentinan, a polysaccharide that induces a toxic inflammatory response. The rash typically appears within 24 to 48 hours after ingestion and resolves spontaneously without specific treatment.^4^
B. Bleomycin induced toxicity – Correct. Bleomycin, a chemotherapy agent, is a well-known cause of flagellate erythema as a dose-dependent cutaneous toxicity. The rash manifests as linear, hyperpigmented streaks, often pruritic, appearing days to weeks after drug administration. Linear hyperpigmentation following the initial erythema is a characteristic feature.^4^
C. Still disease – Correct. It is typically associated with evanescent, salmon-colored rashes, but some cases of flagellate erythema have been reported. This manifestation is thought to be triggered by cytokine release and excoriations induced by pruritus.^4^
D. Pellagra – Incorrect. Resulting from a severe vitamin B3 (niacin) deficiency, it can cause a photosensitive dermatitis, commonly seen as Casal’s necklace, but does not present with flagellate streaks.^5^
E. Sarcoidosis – Incorrect. Cutaneous sarcoidosis typically manifests as lupus pernio, erythema nodosum, papular or plaque-like lesions, but without linear streaks.

## Conflicts of interest

None disclosed.
